# Lyme Carditis Buried Beneath ST-Segment Elevations

**DOI:** 10.1155/2017/9157625

**Published:** 2017-06-21

**Authors:** Basia Michalski, Adrian Umpierrez De Reguero

**Affiliations:** ^1^Division of General Internal Medicine, Medical College of Wisconsin, Milwaukee, WI, USA; ^2^Division of General Internal Medicine, Section of Hospital Medicine, Medical College of Wisconsin, Milwaukee, WI, USA

## Abstract

Lyme disease is caused by the spirochete* Borrelia burgdorferi* and is carried to human hosts by infected ticks. There are nearly 30,000 cases of Lyme disease reported to the CDC each year, with 3-4% of those cases reporting Lyme carditis. The most common manifestation of Lyme carditis is partial heart block following bacterial-induced inflammation of the conducting nodes. Here we report a 45-year-old gentleman that presented to the hospital with intense nonradiating chest pressure and tightness. Lab studies were remarkable for elevated troponins. EKG demonstrated normal sinus rhythm with mild ST elevations. Three weeks prior to hospital presentation, patient had gone hunting near Madison. One week prior to admission, he noticed an erythematous lesion on his right shoulder. Because of his constellation of history, arthralgias, and carditis, he was started on ceftriaxone to treat probable Lyme disease. This case illustrates the importance of thorough history taking and extensive physical examination when assessing a case of possible acute myocardial infarction. Because Lyme carditis is reversible, recognition of this syndrome in young patients, whether in the form of AV block, myocarditis, or acute myocardial ischemia, is critical to the initiation of appropriate antibiotics in order to prevent permanent heart block, or even death.

## 1. Case Presentation

**Figure 1:**
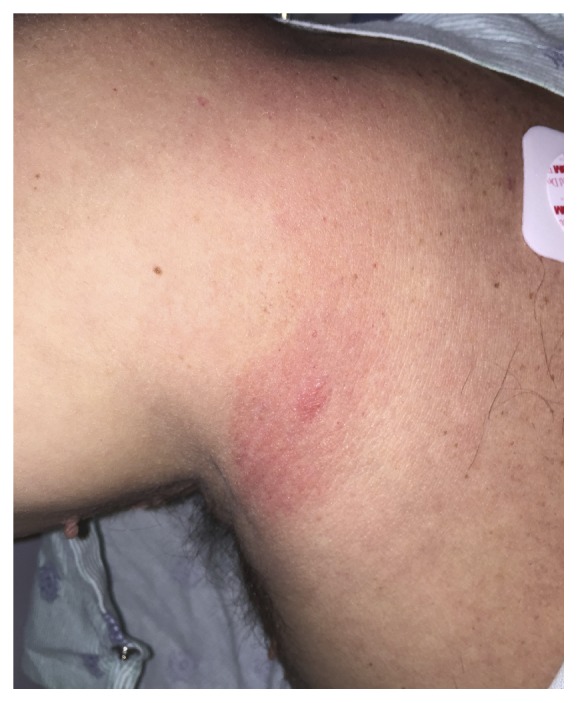
Bulls-eye targetoid rash on patient's right shoulder.

A 45-year-old gentleman with a past medical history significant for hypertension and hyperlipidemia presented to the hospital with left sided chest pain that did not radiate. For the four days prior to admission, patient had a nondocumented fever, diffuse muscle aches, neck and back stiffness, headache, chills, diaphoresis, and nausea. He denied photophobia, blurry vision, sore throat, difficulty breathing, and dizziness. On the morning of hospital admission, he woke up with intense chest pressure, relieved with nitroglycerin given in the Emergency Department. On examination, patient was in no acute distress and alert and oriented to person, place, and time. His temperature was 37.7°C, pulse 95 beats/minute, blood pressure 135/74, respiratory rate 24 per minute, and oxygen saturation was 98% on room air. Cardiac examination demonstrated normal rate, with no murmurs rubs or gallops. Lungs were clear to auscultation bilaterally. Skin exam revealed a 4 × 3 cm erythematous, targetoid bulls-eye lesion on his right shoulder (Figure 1).

**Figure 2:**
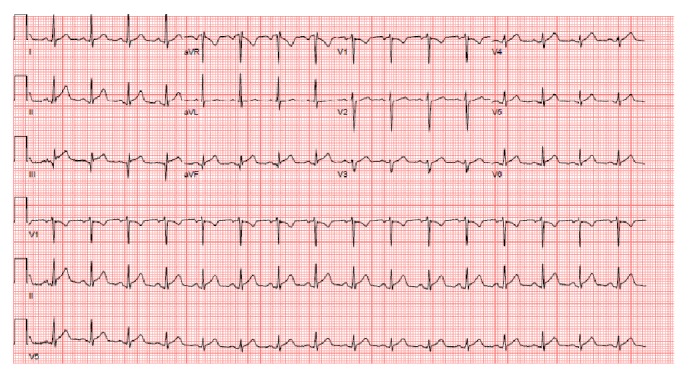
EKG showing normal sinus rhythm, inferior infarct, and ST elevation in inferior leads.

Labs were obtained and were remarkable for creatine kinase 113 U/L (normal 52–336 U/L), CK-MB 4.6 ng/mL (normal < or = 7.7 ng/mL), and troponin T 0.091 ng/mL (normal < 0.01 ng/mL). CBC and LFTs were within normal limits. EKG demonstrated normal sinus rhythm with mild ST elevations in the inferior and high lateral leads and Q waves in III and aVF (Figure 2). Following EKG, he was taken for left heart catheterization. Left coronary angiogram revealed normal coronaries. Transthoracic echocardiogram revealed normal ejection fraction and no wall motion abnormalities or effusion. Further laboratory workup included Lyme titers and confirmatory Western blot analysis which were positive with Lyme IgG/IgM = 1.30 (normal < 0.90).

Three weeks prior to hospital presentation, the patient had gone hunting near Madison. One week prior to admission, he noticed an erythematous lesion on his right shoulder. It had progressed from a small bug bite to a larger, 4 × 3 cm erythematous, painless, nonpruritic, targetoid bull-eyes rash. Because of his constellation of history, arthralgias, and carditis, Infectious Disease was consulted and he was treated empirically for Lyme carditis with ceftriaxone 2.0 grams IV daily. He was also followed with repeat EKG every two days to monitor AV blocks. Prior to discharge, he was switched to oral doxycycline therapy as recommended by Infectious Disease. His symptoms improved and his liver function tests normalized after treatment.

## 2. Discussion

Lyme disease is caused by the spirochete* Borrelia burgdorferi* and is carried to human hosts by infected ticks.* Borrelia burgdorferi* is a member of the spirochete family and survives within a vector, the* Ixodes *tick. There are nearly 30,000 cases of Lyme disease reported to the CDC each year, with 3-4% of those cases reporting Lyme carditis [1]. Lyme disease is notoriously known as the “great imitator” because of its ability to mimic other nonspecific diseases, often leading to delayed or incorrect diagnoses [2]. The goal of this discussion is to identify the pathology and presentation of various forms of Lyme carditis, focusing on the least reported, and perhaps the most rare, presentation, which is of ST-segment elevation myocardial infarction.

There are three described stages of Lyme disease: acute illness, dissemination, and the chronic phase [3]. Acute Lyme disease is characterized by* erythema migrans*. Dissemination phase presents with arthralgias, myalgia, and carditis. In the final and chronic stage of Lyme disease, the host often suffers debilitating arthritis. This discussion will focus on the cardiac implications of Lyme disease inoculation.

As stated earlier, Lyme carditis affects only 3-4% of people with Lyme disease. Interestingly, there is a reported 3 : 1 male : female ratio in patients contracting Lyme carditis [4]. Lyme carditis falls into two distinct categories: conduction disturbances and other complications. The most common conduction disturbance is third-degree atrioventricular (AV) block, with 49% of patients with Lyme carditis reporting a 3rd-degree AV block [4]. These tend to resolve within 6 weeks when inflammation of the myocardium subsides. While AV blocks are the most common presentation of Lyme carditis, other cardiac complications have been described including pericarditis [5], pericardial effusion [6], dilated cardiomyopathy [7], ischemia, and degenerative cardiac valvular disease [8].

Here is a case of a 45-year-old gentleman who presented with left sided chest pain, biomarkers, and electrocardiographic findings consistent with myocardial infarction who was later diagnosed with Lyme carditis. Diagnosis of Lyme carditis can be difficult as the disease can present in a variety of ways. Serologic examination in two steps is the recommended method of disease detection. The first test is a screening assay with an ELISA followed by an immunoassay Western blot. Early stages of the disease can result in false negatives and clinical presentation, with a compelling history for potential tick-borne disease, must be weighed heavily when making a diagnosis.

This case illustrates the importance of thorough history taking and extensive physical examination when assessing a case of possible acute myocardial infarction. Lyme carditis is an uncommon cause of a comparatively common hospital presentation of acute myocardial infarction. Because Lyme carditis is reversible, prompt recognition, whether in the form of AV block, myopericarditis, or ischemia, is of upmost importance to prevent complications such as permanent heart block, or even death.
